# Correction: Hepatitis C virus cascade of care among adults in Sindh province, Pakistan: Findings from 2019–2020 household sero-survey

**DOI:** 10.1371/journal.pgph.0006253

**Published:** 2026-04-06

**Authors:** Tesfa Sewunet Alamneh, Josephine G. Walker, Aaron G. Lim, Ejaz Alam, Saeed Hamid, Graham R. Foster, Naheed Choudhry, M. Azim Ansari, Huma Qureshi, Peter Vickerman

There are errors in the Funding statement. The correct Funding statement is as follows: This work was supported by the NIHR Health Protection Research Unit (HPRU) in Behavioural Science and Evaluation at the University of Bristol (NIHR200877 to AGL and PV); the Wellcome Trust (WT220866/Z/20/Z to JW, AGL, and PV); and the Sir Henry Dale Fellowship jointly funded by the Royal Society and the Wellcome Trust (220171/Z/20/Z to MAA). Additional support was provided by the NIHR (NIHR133208 to JW, AGL, and PV). TSA acknowledges the PhD scholarship from the DESTINE project funded by the NIHR (NIHR133208) using UK international development funding from the UK Government to support global health research. The views expressed in this publication are those of the author(s) and not necessarily those of the NIHR or the UK government. The funders had no role in study design, data collection and analysis, decision to publish, or preparation of the manuscript.
