# Supplementary material for: Hepatitis C virus cascade of care among adults in Sindh province, Pakistan: Findings from 2019–2020 household sero-survey
Source: PLOS Glob Public Health. 2025 Jul 8;5(7):e0004706. doi: 10.1371/journal.pgph.0004706 (PMC12237067; doi:10.1371/journal.pgph.0004706)
Supplement: S1 Text — (DOCX) [file pgph.0004706.s001.docx]

**Hepatitis C virus Cascade of Care among adults in Sindh province, Pakistan: Findings from 2019-2020 household sero-survey**

Tesfa Sewunet Alamneh^1,2^, Josephine G Walker^1^, Aaron G Lim^1^, Ejaz Alam^3^, Saeed Hamid^4^, Graham R Foster^5^, Naheed Choudhry^5^, M. Azim Ansari^6^, Huma Qureshi^7^, Peter Vickerman^1^

^1^Population Health Sciences, Bristol Medical School, University of Bristol, Bristol, UK

^2^Department of Epidemiology and Biostatistics, Institute of Public Health, College of Medicine and Health Sciences, University of Gondar, Ethiopia

^3^Pakistan Health Research Council Research Centre, Karachi, Pakistan

^4^Aga Khan University, Karachi, Pakistan,

^5^Queen Mary University of London, London, UK

^6^Nuffield Department of Medicine, University of Oxford, Oxford, UK

^7^Consultant Gastroenterologist, Doctor Plaza, Clifton Karachi

Of 200 adults that tested Ab-negative in the survey, 84.5% (186/220) reported a negative result from their previous test, whereas 87.0% (100/115) of adults that had a positive HCV-Ab test result in the survey reported a positive result from their previous HCV test (Table A).

Table A. Discordance of self-reported HCV test results and HCV-Ab test results from the 2019-2020 survey.

|  | | HCV-Ab test results in the 2019-2020 survey | | | |
| --- | --- | --- | --- | --- | --- |
|  |  | Negative | | Positive | |
| Self-reported HCV test results | Negative | 186 | 84.5 | 15 | 13.0 |
|  | Positive | 34 | 15.5 | 100 | 87.0 |

Of those adults who reported a previous positive test result, but tested HCV-Ab negative in the survey, 66.7% (22/33) reported previous HCV treatment and 29.4% (10/34) reported their previous test was 5 or more years ago (Table B**)**

Table B. Hepatitis C virus testing, treatment and diagnosis results from the 2019-2020 household sero-survey

| Variable | n | Number | Percent |
| --- | --- | --- | --- |
| Adults tested HCV-Ab positive of those adults tested for HCV-Ab in survey | 3684 | 397 | 10.8% |
| Adults reporting ever being tested of those testing HCV-Ab positive in survey | 397 | 116 | 29.2% |
| Adults reporting ever being diagnosed (self-reported they had a positive test result) of those tested HCV-Ab positive | 397 | 100 | 25.1% |
| Adults tested HCV-RNA of those tested HCV-Ab positive | 397 | 333 | 83.8% |
| Adults having a valid HCV-RNA test result of those tested HCV-Ab positive | 397 | 321 | 80.8% |
| Adults testing HCV-RNA positive of those having a valid HCV-RNA test result | 321 | 204 | 63.4% |
| Adults reporting ever being tested of those tested HCV-RNA positive | 204 | 67 | 32.8% |
| Adults reporting ever being diagnosed (self-reported they had a positive test result) of those tested HCV-RNA positive | 204 | 59 | 28.9% |
| Adults reported receiving HCV treatment of those tested HCV-RNA negative | 117 | 28 | 23.9% |
| Adults with illiterate educational level having discordant results | 136 | 27 | 19.9% |
| Adults with primary educational level having discordant results | 65 | 10 | 15.4% |
| Adults with secondary educational level having discordant results | 111 | 9 | 8.1% |
| Adults with unknown educational level having discordant results | 23 | 3 | 13.0% |
| Adults reporting receiving HCV treatment of those adults that tested HCV-Ab negative result but reported a previous positive test | 33 | 22 | 66.7% |
| Adults reporting not receiving HCV treatment of those adults that tested HCV-Ab negative but reported a previous positive test | 33 | 8 | 24.2% |
| Adults that don’t know their HCV treatment status of those adults that tested HCV-Ab negative but reported a previous positive test | 33 | 3 | 9.1% |

When we restricted our analysis to individuals who have attended at least primary education, the HCV testing coverage was slightly higher as expected, 13.1% (180/1373) among individuals who attended at least primary education versus 9.3% (341/3864) overall. Regarding the cascade of care, from 62 treatment-eligible individuals who attended at least primary education, 53.2% (33/62) reported ever being tested versus 41.2% (95/232) overall, 93.9% (31/33) reported testing positive versus 91.6% (87/95) overall, and 74.2% (23/31) reported receiving HCV treatment versus 69.0% (60/87) overall. Older age (individuals aged 25 to 64 (aOR=7.4, 95%CI: 3.4-15.7) and 65+ years (aOR=4.1, 95%CI:1.3-13.1) compared to individuals aged 18 to 24), having a family history of hepatitis (aOR=3.3, 95%CI:2.1-5.1), blood transfusion (aOR=2.7, 95%CI:1.5-4.9 or HBV vaccination (aOR=8.0, 95%CI:4.5-14.1) were positively associated with HCV ever testing in this reduced sample.
